# Enhanced Photothermal Property of NDI-Based Conjugated Polymers by Copolymerization with a Thiadiazolobenzotriazole Unit

**DOI:** 10.1021/acsmaterialsau.3c00077

**Published:** 2023-10-26

**Authors:** Mingqian Wang, Chia-Yang Lin, Yoshimitsu Sagara, Tsuyoshi Michinobu

**Affiliations:** Department of Materials Science and Engineering, Tokyo Institute of Technology, 2-12-1 Ookayama, Meguro-ku, Tokyo 152-8552, Japan

**Keywords:** Copolymers, organic semiconducting polymers, photothermal conversion, solar steam generation, thiadiazolobenzotriazole

## Abstract

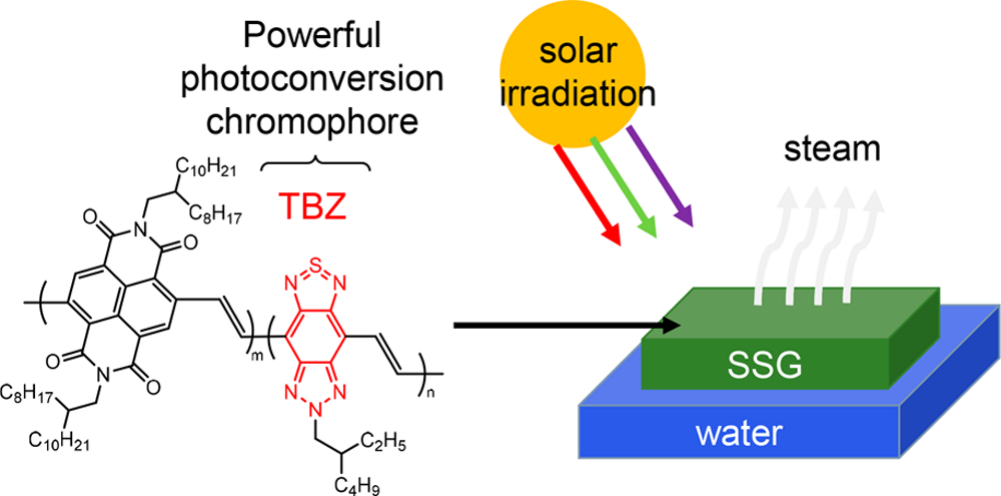

Solar steam generation (SSG) is a promising photothermal technology to solve the global water storage issue. The potential of π-conjugated polymers as photothermal materials is significant, because their absorption range can be customized through molecular design. In this study, naphthalenediimide (NDI) and thiadiazolobenzotriazole (TBZ) were employed as bifunctional monomers to produce conjugated polymers. There are two types of polymers, **P1** and **P2**. **P1** is based on NDI, while **P2** is a copolymer of NDI and TBZ in a ratio of 9:1. Both polymers had high molecular weights and sufficient thermal stability. UV–vis–near-infrared (NIR) absorption spectra revealed that both polymers have large extinction coefficients ascribed to the NDI and TBZ chromophores. Notably, the absorption spectrum of **P2** exhibited a significant red shift compared to **P1**, resulting in a narrow optical bandgap and absorption in the NIR range. This result suggested that **P2** has a higher light absorption than **P1**. Photoluminescence (PL) spectra were measured to elucidate the conversion of the absorbed light into thermal energy. It was found that **P2** has a reduced fluorescence quantum yield as a result of the TBZ unit, signifying a proficient conversion of the photothermal energy. Based on the results, it appears that the **P2** film has a greater photothermal property compared to that of the **P1** film. The surface temperature of the **P2** film reached approximately 50 °C under the investigated conditions. In addition, copolymer **P2** exhibited an impressive SSG efficiency of 72.4% under 1 sun (1000 W/m^2^) irradiation. All the results suggested that narrow bandgap conjugated polymers containing the TBZ unit are highly effective materials for achieving optimal performance in SSGs.

## Introduction

The scarcity of water has emerged as a prevalent concern for society, primarily due to population growth and environmental pollution. Therefore, the task of discovering energy-efficient methods for purifying water is of utmost urgency.^1−8^ Solar energy is an environmentally friendly, stable, and renewable energy source with a wide spectrum and large surface area.^9^ It is considered one of the most promising renewable energy sources with continuous, stable, and high-intensity energy output. Solar energy is thus an ideal power source for water evaporation. Solar energy-based evaporation plays an important role worldwide as an essential component of nature’s water purification systems. For centuries, humans have relied on it as a means of accessing clean water.^10^ Although solar energy is a natural source, it is not efficient in evaporating water, and the utilization of freshwater resources is unable to keep up with the pace of consumption. To address this problem, researchers have begun to focus on efficient, stable, and low-cost technologies that can convert solar energy to thermal energy. Solar steam generation (SSG) is a system that converts solar energy into thermal energy, allowing for the rapid vaporization of water and leaving behind pollutants.^11−15^ Compared to other water purification technologies such as reverse osmosis and submicrometer filtration, SSG is a highly efficient and low-cost technology.

SSG devices largely rely on photothermal materials. In recent years, various photothermal materials, such as carbon-based materials, metals, and inorganic semiconducting materials, have been studied and utilized in SSG.^16−19^ These materials greatly improved the efficiency of the photothermal energy conversion. Organic π-conjugated molecules are also promising for SSG devices because of their tunable bandgap and strong absorption in the near-infrared (NIR) region.^20−23^ Hence, they are regarded as a novel category of photothermal substances. For example, Wang et al. synthesized three small molecules based on the common diketopyrrolopyrrole (DPP) dye as the core unit.^24^ The introduction of end-caps and siloxane side chains to the DPP core improved intramolecular charge transfer (ICT) properties, in addition to hydrophobicity. Based on these findings, it was discovered that the DPP derivatives have a broad light absorption range and lower luminescence due to their stronger ICT properties. Additionally, they exhibited efficient solar energy harvesting and photothermal effects, because of their higher nonradiative decay rates. Meanwhile, the hydrophobic nature of these DPP derivatives facilitates the evaporation of water. As a result, a high evaporation efficiency of 71.8% was achieved at visible light power (∼700 W m^–2^). In the realm of conjugated polymers, Liao et al. have reported a conjugated polymer based on porphyrin and aniline that possesses a unique sponge-like porous structure.^25^ A noticeable solar thermal conversion efficiency of 86.3% was achieved under standard sunlight irradiation. In addition, it was also confirmed that nearly 100% of salt and more than 99.2% of dyes can be removed. The materials based on conjugated polymers have showcased exceptional abilities in desalination and dye decolorization with a high level of performance.

Naphthalenediimide (NDI), a commonly used π-chromophore, possesses excellent thermal stability and is frequently utilized in the synthesis of semicrystalline conjugated polymers.^26−29^ Previous studies have shown that the strategy of linking the NDI with other comonomers through a vinylene spacer is a successful approach to extending the conjugation length and minimizing the dihedral angle between the monomer units.^30−33^ It is worth noting that the NDI moiety tends to be fluorescent which could potentially hinder the efficiency of solar thermal conversion.^34,35^ To the best of our knowledge, the application of NDI-based polymers in SSG has not been explored despite numerous reports on them.

Triply fused aromatic ring structures, as represented by benzobisthiadiazole (BBT), have been attracted as a key component to producing narrow bandgap organic semiconductors.^36^ BBT-based semiconducting polymers were first synthesized by Wudl et al. and were evaluated in ambipolar thin film transistors.^37−39^ Our group has also developed a series of BBT-based semiconducting polymers.^40−43^ In the course of studies, the planarity of polymer backbones could be successfully enhanced by replacing the BBT with a thiadiazolobenzotriazole (TBZ) unit. In addition, the polymer packing orientation was controlled by side-chain engineering, resulting in an edge-on orientation suitable for transistor applications. However, the research direction of these polymers shifted due to their narrow bandgap features. A variety of triply fused aromatic systems, including BBT and TBZ units, was recently employed as near-infrared emitting and photothermal therapy materials.^44−46^

Taking the characteristics of the triply fused aromatic ring systems into account, we now report the effect of the TBZ unit on the photothermal property of NDI-based conjugated polymers. Because of the so-called acceptor-acceptor structure of NDI-TBZ copolymers, the intramolecular electronic interactions are simple, and the effect of the TBZ unit can be quantitatively estimated. NDI-based conjugated polymers were synthesized by Stille polycondensation of aryl dibromide and *trans*-1,2-bis(tributylstannyl)ethylene. The introduction of a vinylene spacer effectively enhanced backbone planarity, which enabled an evaluation of the chromophore effect. Optical and electrochemical properties were measured to investigate the bandgaps of the polymers. Copolymer with the TBZ unit exhibited a narrower bandgap and NIR absorption as compared to the counter NDI-based polymer. Photoluminescence measurements suggested that the TBZ-containing copolymer shows a higher nonradiative decay rate constant and photothermal conversion efficiency. This was also supported by the SSG efficiencies. All these results indicated that conjugated polymers are promising SSG materials, and the TBZ unit can be used to enhance the photothermal conversion efficiency.

## Results and Discussion

### Polymer Synthesis and Characterization

**Scheme 1:**
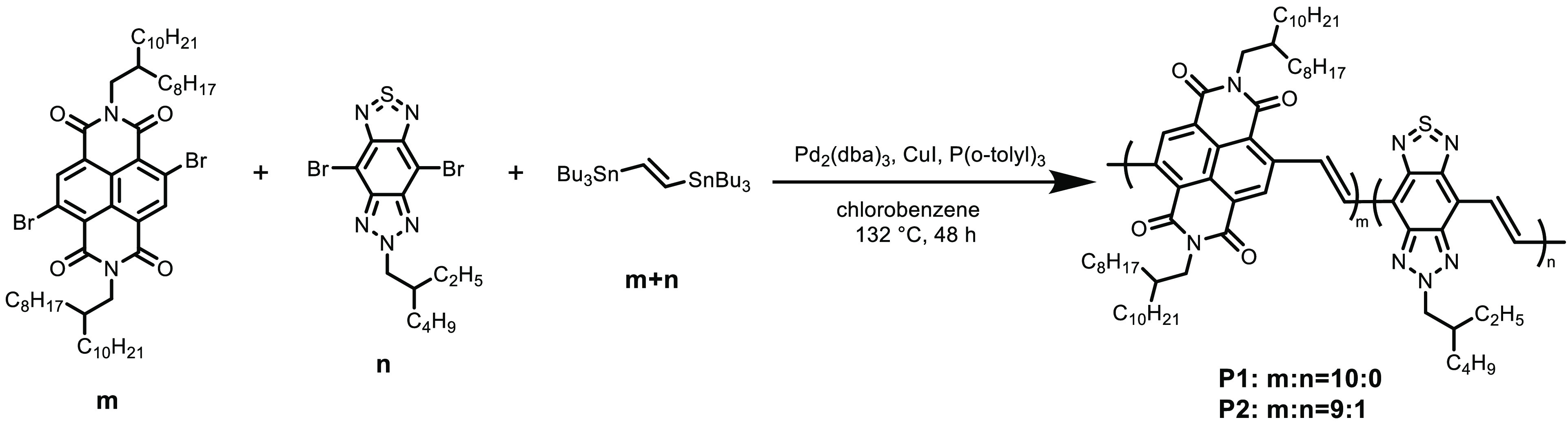
Synthesis of NDI-TBZ Copolymers

NDI-based copolymers were synthesized by Pd/CuI-catalyzed Stille coupling polycondensation according to our previous report (Supporting Information).^28^ We initially synthesized NDI-based polymer **P1** (Scheme 1).^32^ It was shown that **P1** is soluble in common organic solvents such as chloroform, chlorobenzene, and 1,2-dichlorobenzene. Based on this result, we decided to synthesize NDI-TBZ copolymers. Various ratios of NDI to TBZ were tested, and copolymer **P2** with a NDI:TBZ (*m*:*n*) ratio of 9:1, which has a solubility similar to that of **P1**, was successfully obtained. It should be noted that when the TBZ content exceeded 20 mol %, the resulting copolymers became insoluble in any organic solvents. This result suggested that side-chain alkyl groups have a significant impact on the polymer solubilities and that poly(arylenevinylene)s have highly planar backbone structures due to the conformational lock effect of the intramolecular hydrogen bonding.^33^ Both polymers were purified by Soxhlet extraction with methanol, acetone, and hexane to remove small molecular weight fractions, and the dichloromethane soluble fractions were reprecipitated into methanol and characterized by ^1^H NMR (Figures S1 and S2). Gel permeation chromatography (GPC) measurements using polystyrene as the standard sample and 1,2-dichlorobenzene as the eluent at 40 °C revealed that **P2** has a number-average molecular weight (*M*_n_) of 43 kg/mol with a polydispersity index (PDI) of 4.3, while **P1** shows the *M*_n_ of 36 kg/mol and PDI of 5.6 (Table 1 and Figure S3).

**Table 1:** Molecular Weights and Thermal Properties of Polymers

polymer	*M*_n_ (kg/mol)^a^	PDI^b^	*T*_d_ (°C)^c^
**P1**	36	5.6	250
**P2**	43	4.3	385

a Number-average molecular weight.

b Polydispersity index (*M*_w_/*M*_n_).

c Temperature at which 5% weight loss occurred.

To investigate the thermal properties of the two polymers, thermogravimetric analysis (TGA) was performed (Figure S4a). As summarized in Table 1, both **P1** and **P2** polymers exhibited good thermal stabilities with 5% weight loss temperatures (*T*_d_) of 250 and 385 °C, respectively. Differential scanning calorimetry (DSC) measurements suggested that **P1** has no well-defined transition peaks in the temperature range 25–200 °C, while **P2** exhibits an exothermic transition at 260 °C (Figure S4b and c).

### Optical and Electrochemical Properties

**Figure 1:**
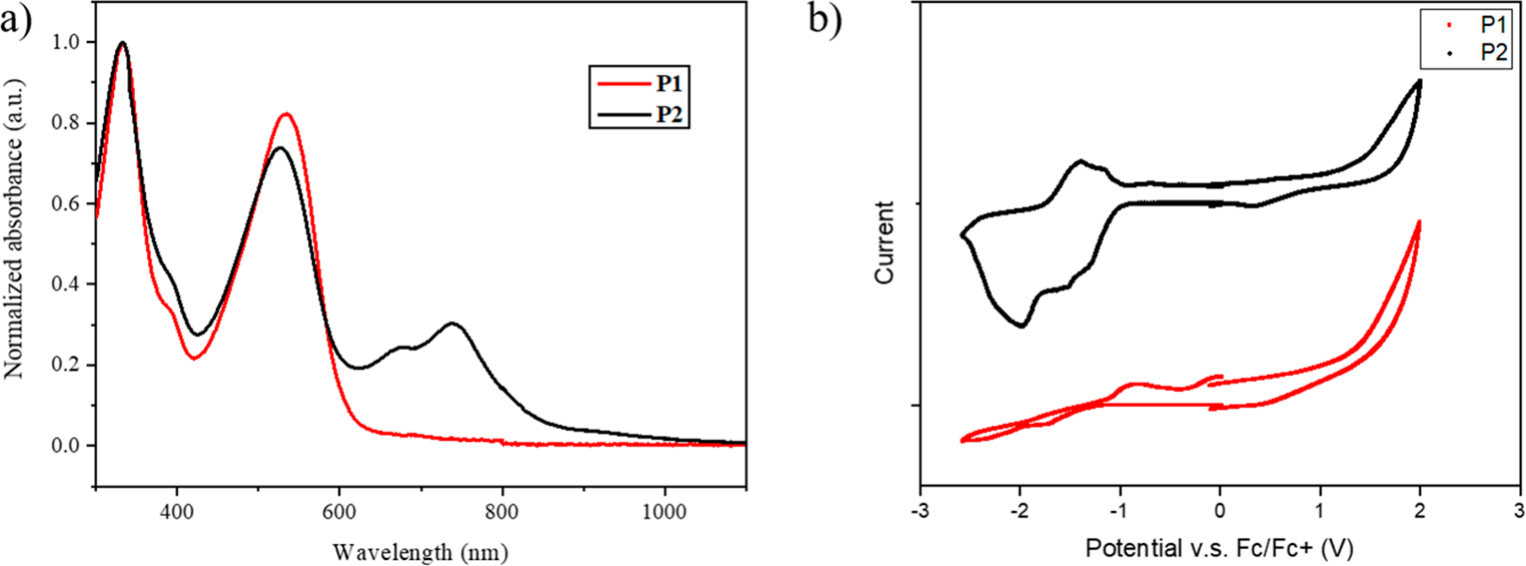
(a) UV–vis-NIR absorption spectra and (b) CV curves of the **P1** and **P2** film.

To reveal the optical properties of the two polymers, ultraviolet (UV) – vis near-infrared (NIR) spectra were measured. The absorption spectra of **P1** and **P2** in chloroform solutions or thin-film states, prepared by spin-coating on a quartz substrate, were measured (Figures 1a and S5) and the relevant data are summarized in Table 2. **P1** and **P2** showed similar absorption profiles with peak tops of about 330 and 550 nm in the range 300–600 nm, which most likely originate from the NDI-based backbones. However, only **P2** displayed new absorption peaks at around 730 nm in both dilute chloroform solution and thin film. Based on the chemical structures, these peaks are attributed to the TBZ unit. The thin film spectrum showed two well-defined peaks in this region, while the solution spectrum displayed a broad single peak. This result suggests that **P2** has strong intermolecular interactions in the solid state. The extinction coefficients of one repeat unit (ε) were calculated from the absorbance of the solution spectra at the concentration of 6 × 10^–6^ M (Figure S5). Both polymers had ε of >200 000 M^–1^ cm^–1^ at 330 and 550 nm, suggesting the powerful light absorbing properties of the conjugated backbones, and **P2** had an additional low energy absorption due to the TBZ unit. The thin film absorption spectrum of **P2** displayed a considerable red shift as compared to that of **P1**, with the onset absorption (λ_onset_) shifting from 624 nm in **P1** to 894 nm in **P2**. The resulting estimated optical bandgap (*E*_g_^OPT^) of **P2** was 1.38 eV, which is significantly lower than that (1.98 eV) of **P1**. More importantly, the absorption band of **P2** extends into the NIR region. This is attributed to the synergy of the highly planar backbone structure and triply fused aromatic ring. The NIR region is reported to account for approximately 52% of the radiation spectrum emitted by the sun.^47,48^ Therefore, as **P2** has the ability to absorb NIR light, it can capture a greater amount of solar radiation compared to **P1**. As a result, more thermal energy is converted and released; improving the SSG performance is crucial, and this plays a significant role.

**Table 2:** Optical and electrochemical properties of **P1** and **P2**

polymer	λ_max,sol_ (nm)^a^	ε (M^–1^ cm^–1^)^b^	λ_max,film_ (nm)^c^	λ_onset,film_ (nm)^c^	*E*_g_^opt^ (eV)^d^	*E*_LUMO_ (eV)^e^	*E*_HOMO_ (eV)^f^
**P1**	519	213 000	534	624	1.98	–3.51	–5.49
**P2**	727	52000	737	894	1.38	–3.77	–5.15

a Longest wavelength absorption maxima of the polymers in chloroform solution (6 × 10^–6^ M).

b Monomer repeat unit extinction coefficient.

c Longest wavelength absorption maxima and onset absorption of the polymer thin films spin-cast on a quartz substrate.

d Estimated from the onset absorption of the film using the equation *E*_g_^opt^ = 1240/λ_onset_.

e Estimated from the onset reduction potentials of the polymer thin films in CH_3_CN with 0.1 M (*n*C_4_H_9_)_4_NClO_4_ at a scan rate of 0.1 V s^–1^.

f Calculated by *E*_HOMO_ = *E*_LUMO_ – *E*_g_^opt^.

The HOMO and LUMO energy levels of **P1** and **P2** were estimated by cyclic voltammetry (CV) and optical bandgaps. Both polymers exhibited reduction peaks (Figure 1b). The LUMO energy level of **P2** (−3.77 eV), estimated from the onset reduction potential, was deeper than that of **P1** (−3.51 eV). This can be attributed to the stronger electron-accepting ability of the polymer backbone with the TBZ unit. The HOMO energy level of **P1** was calculated from the LUMO energy level and optical bandgap. Due to the introduced TBZ unit, **P2** showed a weak oxidation peak with an onset potential of 0.43 V. The HOMO levels of **P1** and **P2** were −5.49 and −5.15 eV, respectively.

In an ideal photothermal material, the excited energy should be fully converted into nonradiative relaxation to provide a higher light-to-heat energy conversion rate.^49^ Therefore, photothermal materials should be nonradiative. To further investigate this issue, we measured the photoluminescence (PL) of compounds **P1** and **P2**.

**Figure 2:**
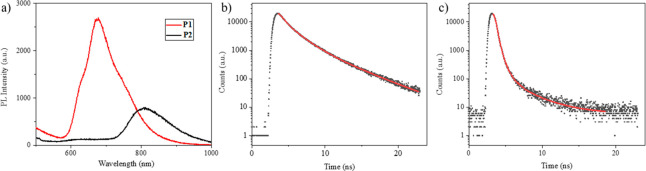
(a) Photoluminescence spectra of the **P1** and **P2** solids (excited at 385 nm). Fluorescence lifetimes of (b) **P1** and (c) **P2** solids.

The excitation wavelength of two polymers was set at 385 nm. The emission peaks of **P1** and **P2** were observed at 678 and 807 nm, respectively (Table 3). The peak intensity of **P2** was significantly lower than that of **P1**. As a result, the radiative properties of **P2** were estimated to be lower than those of **P1**. Subsequently, the fluorescence quantum yields (Φ_f_) were measured. **P1** showed a higher quantum yield of 0.06 than **P2** (<0.01). This result was further supported by fluorescence lifetime measurements. The fluorescence decay curves and their fitting results of the **P1** and **P2** solids are shown in Figure 2b and c, respectively. The calculated fluorescence lifetime (τ) of **P1** was 2.12 ns, which was larger than that of **P2** (0.18 ns).

**Table 3:** Summary of Photoluminescence Properties

polymer	λ_em_ (nm)^a^	Φ_f_^b^	τ (ns)^c^	*k*_r_ (10^8^ s^–1^)^d^	*k*_nr_ (10^8^ s^–1^)^e^
**P1**	678	0.06	2.12	0.29	4.43
**P2**	807	<0.01	0.18	0.41	54.3

a Peak of emission wavelength.

b Quantum yield.

c Fluorescence lifetime.

d Radiative decay rate constant.

e Nonradiative decay rate constant.

Based on the Φ_f_ and τ values, the radiative decay rate constant (*k*_r_) and nonradiative decay rate constant (*k*_nr_) were estimated by the following equations:
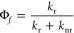
1
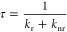
2It is clearly shown that **P2** has a significantly higher *k*_nr_ of 5.43 × 10^9^ s^–1^ than **P1** (4.43 × 10^8^ s^–1^), indicating a better photothermal conversion efficiency. This result is consistent with the other photophysical properties.

### DFT Calculations

**Figure 3:**
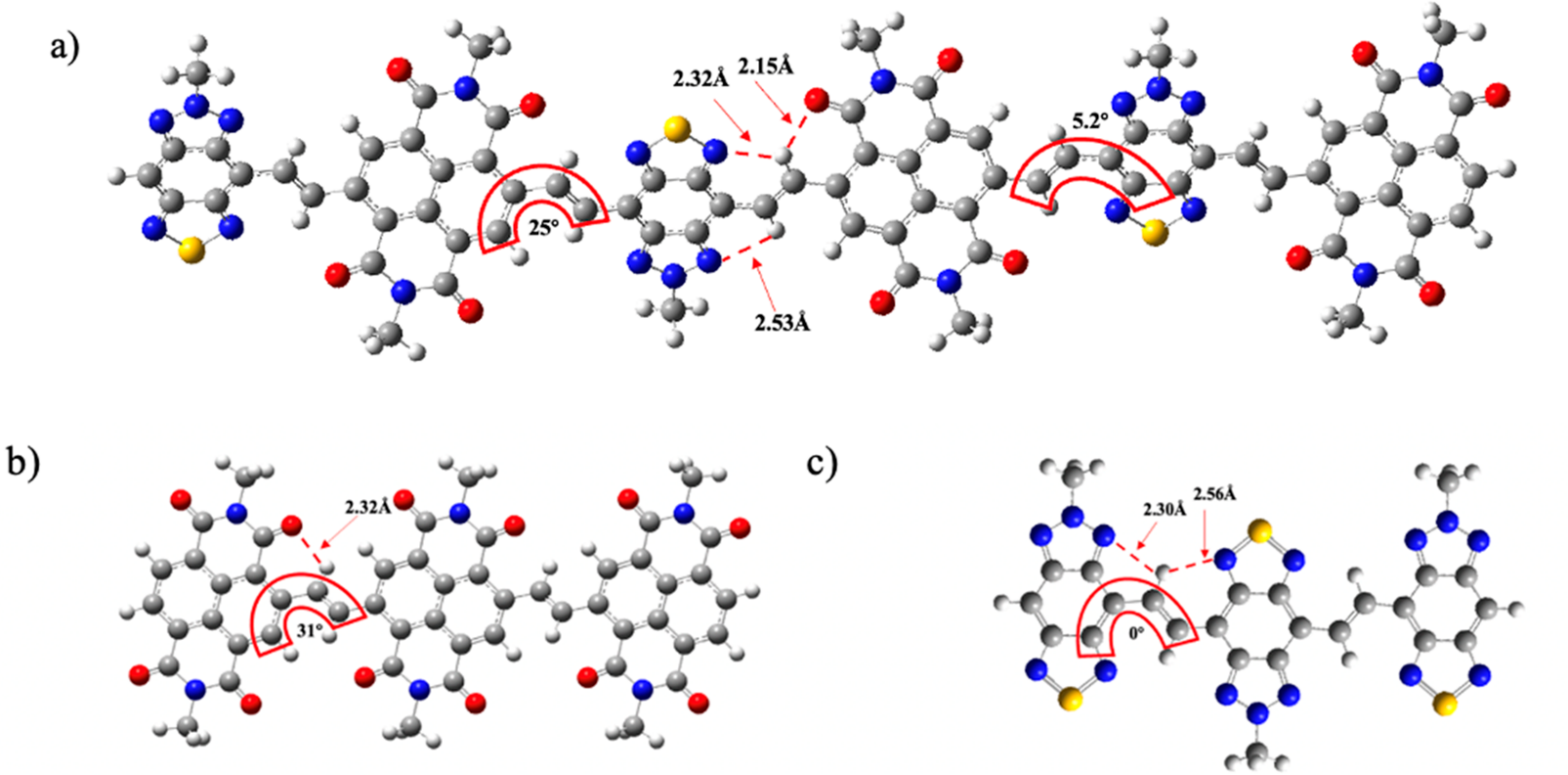
DFT analysis of (a) 3 repeat units of NDI-TBZ, (b) 3 repeat units of NDI, and (c) 3 repeat units of TBZ.

To estimate the polymer backbone geometry, DFT calculations were performed for the possible trimer units of **P1** and **P2**. To reduce the calculation time, we simplified alkyl side chains to methyl groups. To optimize the backbone geometry of two polymers, three trimeric oligomer models with different monomer combinations were created (Figure 3). The molecular backbone of **P1** consists solely of the NDI chromophore, while **P2** has three variants, namely, (TBZ-NDI)_3_, (NDI)_3_, and (TBZ)_3_. To verify the presence of intramolecular hydrogen bonds between the H atom and the O and N atoms, we estimated the distances between these atoms in each unit for the optimized structures. In the case of (NDI-TBZ)_3_, the O···H distance was 2.15 Å, which is shorter than the sum of the O and H van der Waals radius of 2.50 Å. On the other hand, the distances between the N and H atoms were 2.32 and 2.53 Å, which are shorter than the sum of the N and H van der Waals radius of 2.75 Å.^50^ These results prove the existence of hydrogen bonding. The dihedral angle between the NDI and vinylene spacer was 25°, while that between TBZ and vinylene spacer was 5.2°. In the (NDI)_3_ repeat unit, the distance between the O atom of the carbonyl group and the H atom of the vinylene spacer was 2.32 Å, which is also shorter than the sum of the O and H van der Waals radius of 2.50 Å. The dihedral angle between the repeat units was 31°. Finally, for the (TBZ)_3_ repeat unit, the N···H distances were 2.56 and 2.30 Å, again shorter than the sum of the N and H van der Waals radius. In this model, extremely small torsions (dihedral angles ∼ 0°) were achieved. This series of calculations suggests that **P2**, as a ternary random copolymer, has a higher backbone planarity than homopolymer **P1**.

### Fabrication of Photothermal Polymer Membranes and Their Surface Morphology

**Figure 4:**
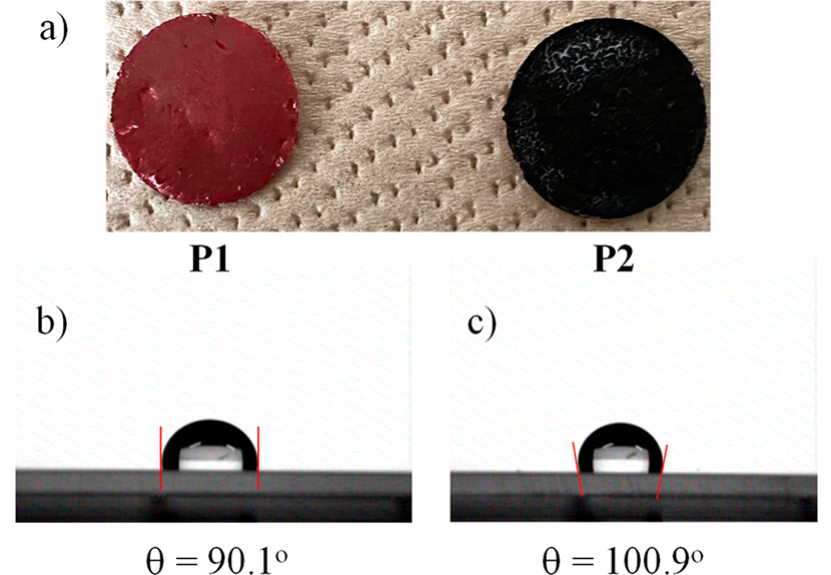
(a) Photothermal polymer membranes of **P1** and **P2**. Water contact angles on (b) **P1** (left) and (c) **P2** (right).

SSG efficiencies were evaluated by fabricating photothermal polymer membranes (Figure 4a). A commercial filter paper (Kiriyama, No.5B, diameter 21 mm) was used as a substrate. **P1** or **P2** was dissolved into chloroform at a concentration of 3 mg/mL. After sonication for 5 min, the solution was dropwise poured into 50 mL of methanol to form precipitates. After stirring the mixture for 20 min, the suspension was deposited on the filter paper by suction filtration. The filter papers with deposited **P1** or **P2** were eventually vacuum-dried overnight at room temperature to produce the photothermal polymer membranes. In this way, the polymers were better dispersed on the membrane surface and were firmly anchored to the filter paper surface.

To investigate the hydrophobicity of the two photothermal membrane surfaces based on **P1** and **P2**, the contact angles of water and glycerol were measured and the surface energy data are summarized in Table 4. The membranes of **P1** and **P2** have water contact angles of 90.1° and 100.9°, respectively (Figure 4b and c), suggesting that both polymers are more or less hydrophobic. The underside of the membranes is a filter paper, a hydrophilic material that easily absorbs water (contact angle ∼ 0°; see Figure S6). Photothermal membranes are usually designed with a hydrophilic layer at the bottom for efficient water uptake and transport and a hydrophobic layer at the top to facilitate water evaporation. The membrane design of this study is very simple but meets this requirement. The contact angles of glycerol were then measured, and the surface energies were calculated (see Supporting Information). The glycerol contact angles of the **P1** and **P2** membranes and filter paper were 84.6°, 90.2°, and 46.7°, respectively (Figure S5 and S6). In general, the higher the surface energy, the easier it is to wet the interface. The surface energies of the **P1** and **P2** membranes were 17.2 and 19.1 mN/m, respectively. This indicates that both **P1** and **P2** are difficult to wet. Compared to **P1** and **P2**, the surface energy of the filter paper was close to 100 mN/m and very wettable. This result again indicates that the fabricated photothermal membranes are suitable for SSG applications.

**Table 4:** Surface Energy of the Photothermal Membrane Surfaces

	θ_water_ (deg)^a^	θ_glycerol_ (deg)^b^	γ (mN/m)^c^
**P1**	90.1	84.6	19.1
**P2**	100.9	90.2	17.2
Filter paper	0	46.7	98.9

a Water contact angle.

b Glycerol contact angle.

c Surface energy.

**Figure 5:**
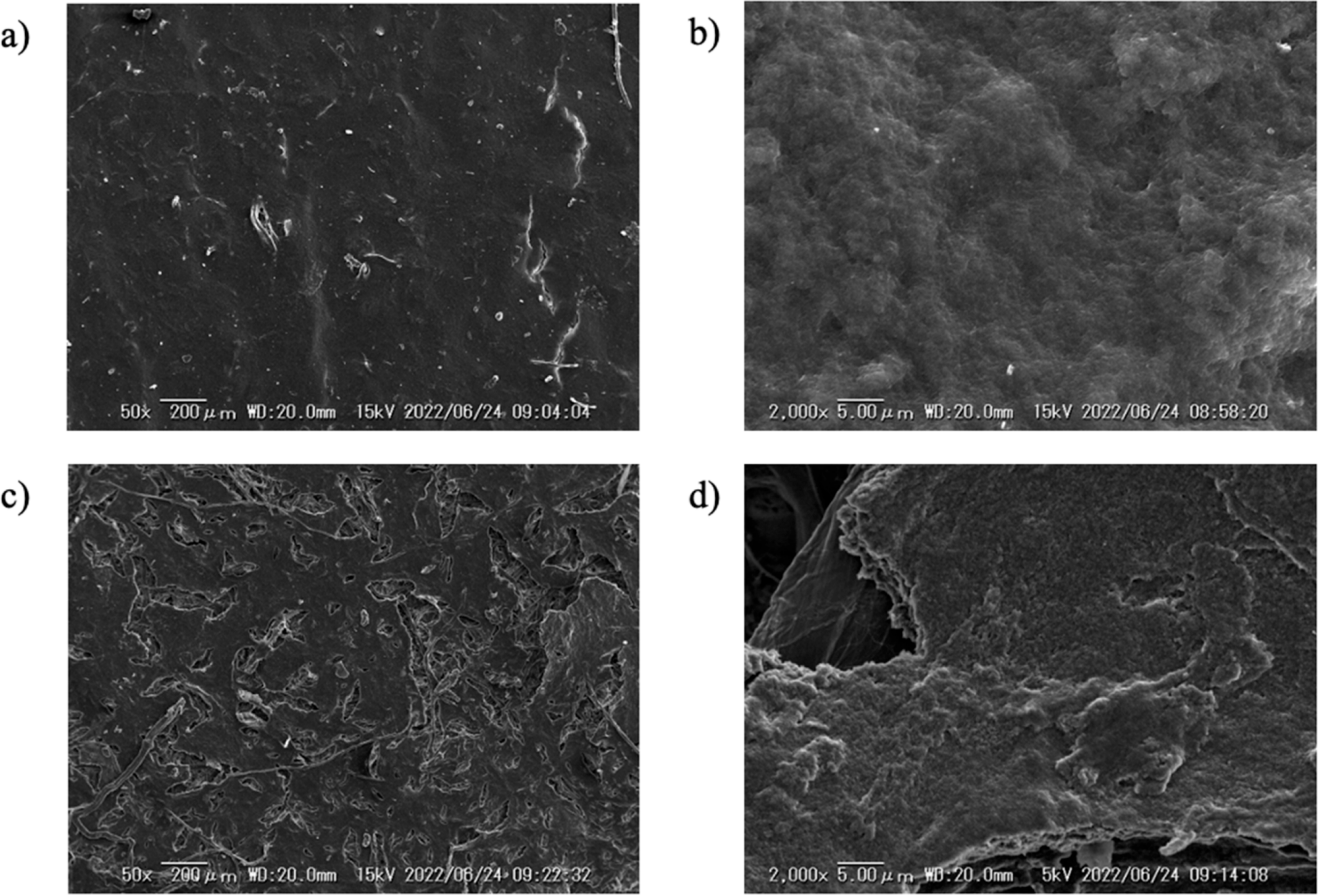
SEM images of **P1** ((a) 50× magnification, (b) 2000× magnification) and **P2** membranes ((c) 50× magnification, (d) 2000× magnification) deposited on a filter paper.

Next, the surface morphology of the **P1** and **P2** membranes was visualized by SEM observations. It was shown that the microstructure of **P1** is continuous and no pore-like structures are present (Figure 5a and b). In contrast, the microstructure of **P2** was rough with some holes, and it was anticipated that these holes facilitate water evaporation (Figure 5c and d). The surface morphology predicts a higher evaporation efficiency for the **P2** membrane.

### SSG Efficiency

**Figure 6:**
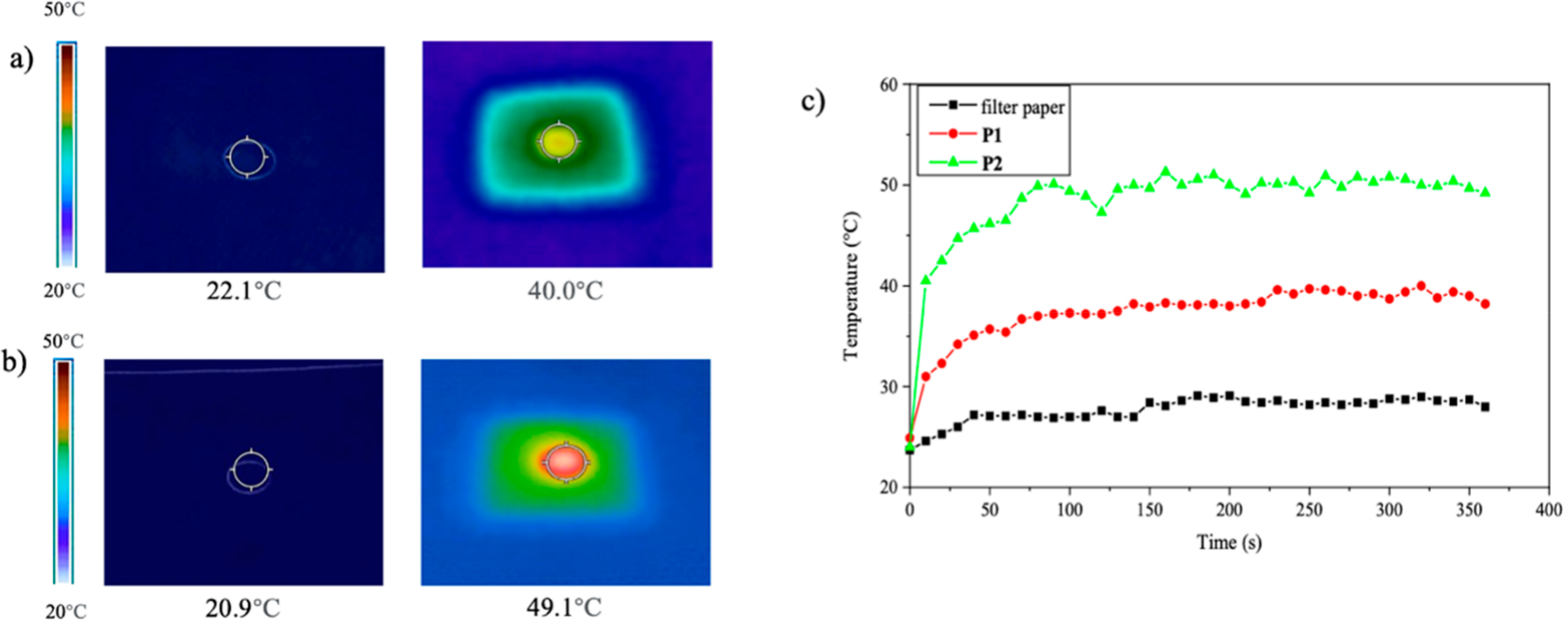
Thermal images of (a) **P1** and (b) **P2** membranes and (c) time-dependent temperature changes of the **P1** and **P2** membranes and filter paper in the dry state under simulated sunlight irradiation.

In order to investigate the SSG properties of the two polymers, temperature changes of the photothermal membranes were recorded with an infrared thermography camera. The thermal images of the photothermal membrane surfaces of **P1** and **P2** are shown in Figure 6a and b, respectively. Solar irradiation clearly increased the surface temperature. In addition, time-dependent temperature changes in the photothermal membranes were measured in the dry states under simulated sunlight irradiation, and the plots were compared to those of filter paper without any conjugated polymers (Figure 6c). Both photothermal membranes of **P1** and **P2** displayed photothermal conversion within minutes. On the other hand, the photothermal conversion of a filter paper, a control sample, did not clearly occur. The saturated surface temperatures of the **P1** and **P2** membranes were 40 and 49 °C, respectively. The difference between the **P1** and **P2** membranes can be explained by the absorption spectra and nonradiative decay constants (vide supra). In other words, the low energy band centered at 737 nm in **P2** and the *k*_nr_ of **P2** (54.3 × 10^8^ s^–1^), which is significantly larger than that of **P1** (4.43 × 10^8^ s^–1^), contribute to the higher surface temperature.

**Figure 7:**
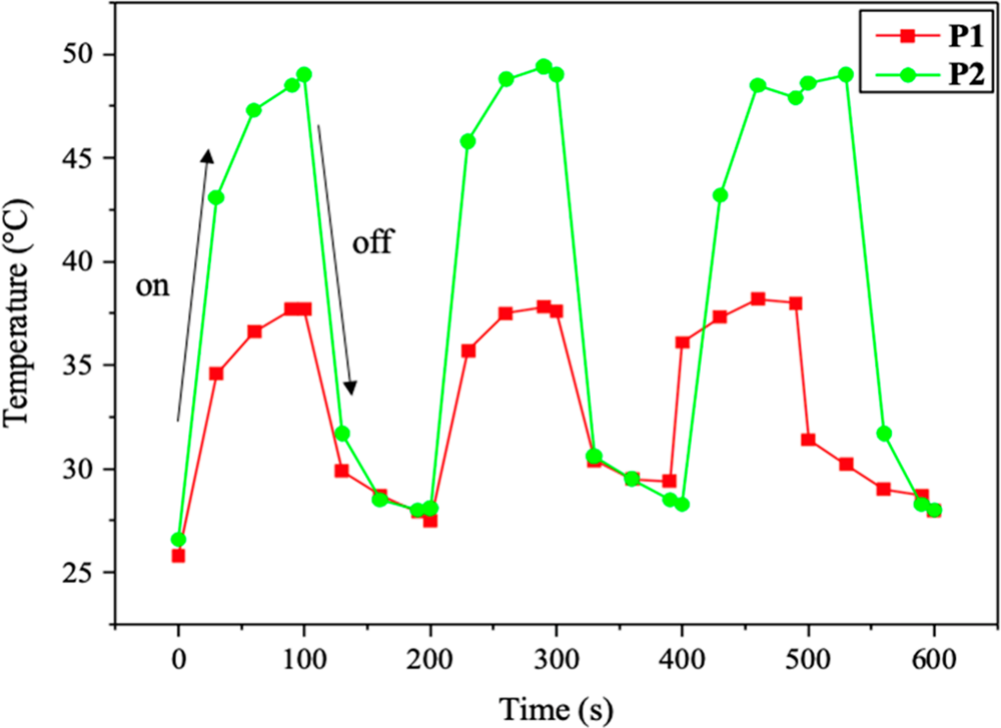
Temperature changes of the photothermal membranes in the dry state under simulated sunlight (3 cycles of sunlight irradiation on/off).

To confirm the reversibility and photostability of the **P1** and **P2**-based photothermal membranes, the simulated solar radiation was turned on for 100 s then turned off for 100 s, and this was repeated for three cycles (Figure 7). The results showed that the temperature changes for the three cycles were nearly identical.

The same repeated experiment was performed in the wet state. Wet conditions were prepared by filling a Petri dish with water and placing the photothermal membranes on top of a sponge that absorbs water. It was observed that the maximum surface temperatures in the wet conditions are lower than those in the dry conditions (Figure S7). Even in the wet state, **P2** exhibited a surface temperature of 36.0 °C, higher than that of **P1** (30.2 °C).

**Figure 8:**
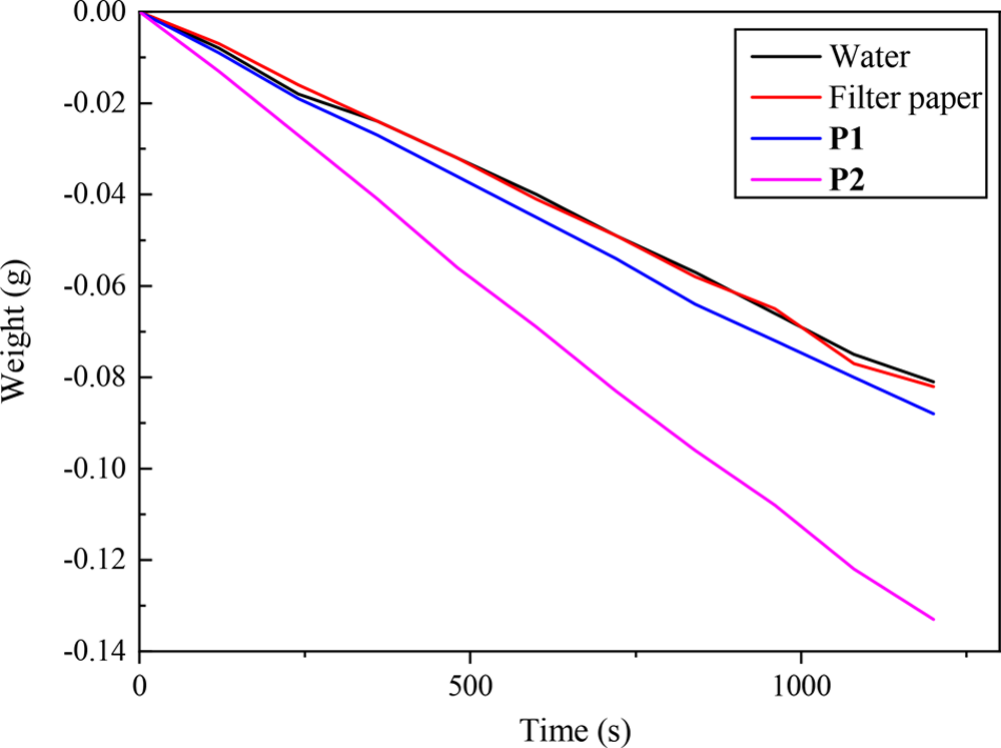
Water weight loss of the photothermal membranes based on **P1** and **P2** under irradiation with simulated sunlight for 1200 s. For comparison, the same experiments were performed for the setups with and without filter paper.

To measure the water vapor generation capacity of the developed photothermal membranes, the weight change of water was recorded (Figure 8). After the photothermal membranes fabricated on a sponge were immersed in water, they were placed on an electronic balance, and the simulated sunlight was used to illuminate the membrane surface. The weight changes during simulated sunlight irradiation were recorded over 1200 s. Water evaporation rates in the dark and under irradiation were also recorded, and the results are shown in Figure 8. The larger weight loss in the **P2** membrane indicates that **P2** achieved a higher evaporation rate than **P1**. This also proves the higher photothermal conversion capability of **P2**. It is noticeable that a small amount of the NIR-absorbing unit of TBZ can dramatically enhance the photothermal conversion efficiency.

**Figure 9:**
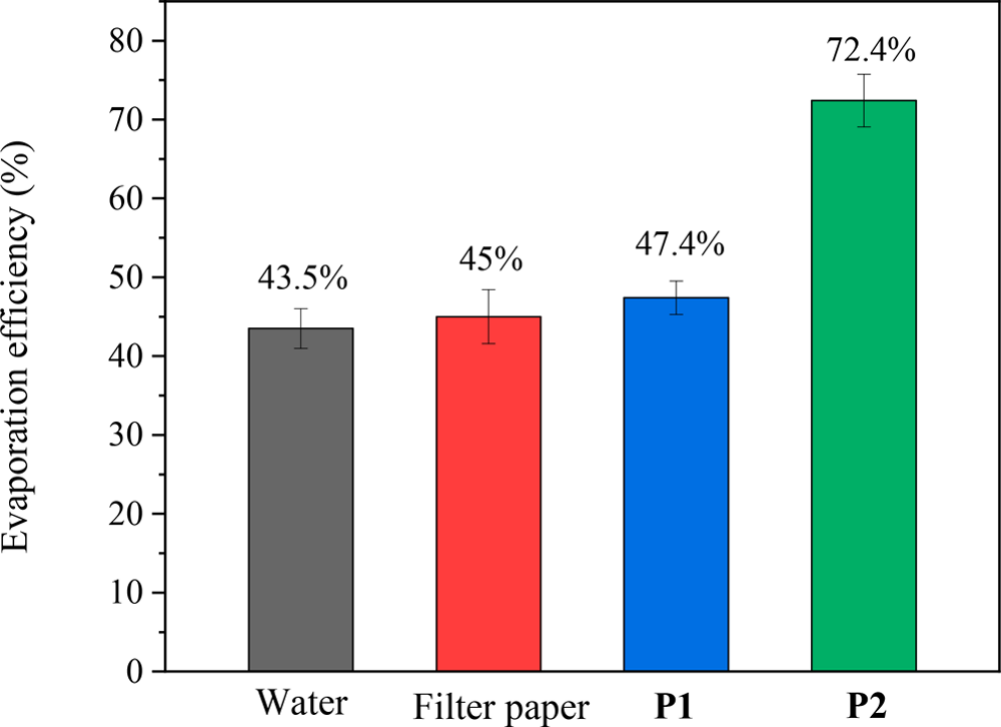
Evaporation efficiencies of bulk water, filter paper, and **P1** and **P2** membranes.

The evaporation efficiencies were estimated based on the water’s evaporation energy and the simulated sunlight’s input energy (see Supporting Information). Under 1 solar (1000 W/m^2^) irradiation, the photothermal membrane based on **P2** exhibited the highest evaporation efficiency of 72.4% (Figure 9). On the other hand, the membrane based on **P1** showed 47.4%, which was slightly higher than that of the filter paper (control sample). This is highly related to the fact that **P1** cannot absorb light in the NIR region and has a film morphology that is unsuitable for water evaporation. In addition, repeated experiments were conducted to understand the stability and reusability of the photothermal membranes. After each measurement, the photothermal membranes and filter paper were completely dried. The results of the repeated experiments did not significantly differ from those of the first experiment, ensuring high stability and reusability of the **P1** and **P2**-based membranes (Figure S8).

## Conclusions

In summary, two NDI-based conjugated polymers, **P1** and **P2**, were designed and synthesized. When the TBZ unit was copolymerized, the resulting polymer **P2** produced an NIR absorption band. In order to reveal the effect of the TBZ unit on photothermal properties, a series of experiments and computational calculations were performed. The photoluminescence kinetic study revealed that **P2** has a greater nonradiative decay rate constant. In addition, the surface morphology study suggested that the **P2** membrane was inhomogeneous as compared to the **P1** membrane. This was beneficial for water transport and evaporation. Based on these facts, solar steam generation efficiencies were evaluated. The result again supported the high potential of the **P2** membrane for SSG applications. It is remarkable that only 10 mol % of the TBZ unit dramatically improved the SSG performance of the NDI-based polymer. The triply fused aromatic ring units, as represented by BBT and TBZ chromophores, have been intensively investigated as NIR dyes for photoacoustic imaging and photothermal therapy.^51^ This study demonstrates that SSG can be added to these application areas.

## Experimental Section

### General Measurements

Nuclear magnetic resonance (NMR) spectra were obtained on a JEOL model 400 YH (400 MHz) spectrometer at room temperature. All chemical shifts were reported in parts per million (ppm). Deuterated chloroform was used as the solvent. ^1^H NMR chemical shifts were referenced to CDCl_3_ (7.26 ppm). Coupling constants (J) are given in Hz. The resonance multiplicity was described as s (singlet) and m (multiplet).

Gel permeation chromatography (GPC) was measured by using a JASCO GULLIVER 1500 instrument equipped with a pump (PU-2080 Plus), an absorbance detector (RI-2031 Plus), and two Shodex GPC KF-803 columns (8.0 mm I.D. × 300 mm L) using *o*-dichlorobenzene as the eluent at the flow rate of 0.5 mL min^–1^ at 40 °C. The molecular weights were calculated based on a calibration curve using polystyrene standards.

Ultraviolet–visible-near-infrared (UV–vis-NIR) spectra were recorded on a JASCO V-670 spectrophotometer in diluted chloroform solutions and in spin-cast thin film states on quartz substrates.

PL spectra were recorded with a JASCO FP-6500 spectrophotometer. The fluorescence lifetimes of the **P1** and **P2** films were measured with a Hamamatsu Photonics Quantaurus-Tau. Quantum yields were measured by a Hamamatsu Photonics Quantaurus-QY.

Thermogravimetric analyses (TGA) were carried out on a Rigaku TG8120 instrument, and differential scanning calorimetry (DSC) measurements were performed on a Rigaku DSC8230 instrument. Both measurements were done under N_2_ flow at the scan rate of 10 °C min^–1^.

The electrochemical measurements were carried out by cyclic voltammetry (CV) using a BAS electrochemical analyzer, model 612C, at room temperature in a classical three-electrode cell. The working, reference, and auxiliary electrodes were a glassy carbon electrode, Ag/AgCl/CH_3_CN/*n*-Bu_4_NClO_4_, and a Pt wire. The polymer films for the electrochemical measurements were drop cast on a glassy carbon electrode from 3 mg mL^–1^ solutions (chloroform). For calibration, the redox potential of ferrocene/ferrocenium (Fc/Fc^+^) was measured under the same conditions, and it was located at 0.10 V versus the Ag/AgCl electrode. Assuming that the redox potential of Fc/Fc^+^ has an absolute energy level of −4.80 eV to vacuum, the HOMO and LUMO energy levels of the polymers were calculated according to the following equations:
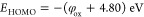

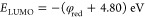
where φ_ox_ is the onset of the oxidation peak and φ_red_ is the onset of the reduction peak.

Contact angles were measured by a Surface Electro Optics Phoenix 150/300 Contact Angle Analyzer. The surface free energy was estimated by the following equations:^52^
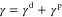

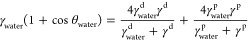

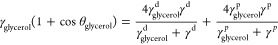
where γ is the total surface free energy, γ^d^ and γ^p^ are the dispersion and polar components of surface free energy, respectively. γ_water_ and γ_glycerol_ are the total free energy of water and glycerol, respectively, and γ_water_^d^, γ_water_^p^, γ_glycerol_^d^, and γ_glyceriol_^p^ are their dispersion or polar components. θ_water_ and θ_glycerol_ are measured contact angles of water and glycerol droplets on films, respectively.

### Characterization of SSG Efficiency

The simulated sunlight was generated by a PEC-L11 Solar Simulator. The energy density of simulated sunlight was measured with a CEM DT-1307 solar power meter. The temperature change of the SSG surface was measured by using an FLIR C5 camera.

The evaporation efficiency (η) was calculated by the following equations:^24^
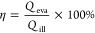

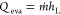

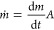
where *Q*_eva_ is the total energy of the evaporated water, *Q*_ill_ is the power density of light illumination, *ṁ* is the total mass loss from evaporation over a specific period, *h*_L_ is the latent heat of vaporization of water (∼2260 kJ kg^–1^), d*m*/d*t* is the evaporation rate from the weight loss measurement after subtracting the amount evaporated under dark conditions, and *A* is the total area of the photothermal membrane.

### Synthesis of the Polymers

All reagents were purchased from Tokyo Chemical Industry (TCI), Kanto Chemical Co., Ltd., Wako Pure Chemical Industries, and Sigma-Aldrich.

### Synthesis of P1

A mixture of 4,9-dibromo-2,7-bis(2-octyldodecyl)benzo[*lmn*][3,8]phenanthroline-1,3,6,8(2*H*,7*H*)tetraone (148 mg, 0.150 mmol), Pd_2_(dba)_3_ (4.60 mg, 5.02 μmol), P(*o*-tolyl)_3_ (6.00 mg, 0.0197 mmol), CuI (3.80 mg, 0.0200 mmol), and *trans*-1,2-bis(tributylstannyl)ethylene (91.0 mg, 0.150 mmol) in chlorobenzene (5 mL) was placed in a Schlenk tube in an argon atmosphere glovebox. The tube was then capped and stirred at 132 °C for 48 h. After cooling to room temperature, the reaction mixture was poured into methanol. HCl (1N, 10 mL) was prepared and slowly added dropwise to the methanol solution. After the mixture was stirred for 20 min, the precipitate was collected by filtration and purified with Soxhlet extraction using methanol, acetone, hexane, and dichloromethane. The dichloromethane soluble fraction was concentrated and reprecipitated into methanol, yielding a red solid (134 mg, yield 91.2%). ^1^H NMR (400 MHz, CDCl_3_) δ 9.18 (s, 1m H), 9.04 (s, 1m H), 4.25 (s, 4m H), 2.19 (s, 2m H), 1.23 (m, 64m H), 0.82 (s, 12m H) ppm.

### Synthesis of P2

A mixture of 4,9-dibromo-2,7-bis(2-octyldodecyl)benzo[*lmn*][3,8]phenanthroline-1,3,6,8(2*H*,7*H*)tetraone (130 mg, 0.140 mmol), 4,8-dibromo-6-(2-ethylhexyl)-[1,2,5]thiadiazolo[3,4*f*]benzotriazole (7.00 mg, 0.0200 mmol), Pd_2_(dba)_3_ (5.00 mg, 5.46 μmol), P(*o*-tolyl)_3_ (6.00 mg, 0.0197 mmol), CuI (4.00 mg, 0.0210 mmol), and *trans*-1,2-bis(tributylstannyl)ethylene (91.0 mg, 0.150 mmol) in chlorobenzene (5 mL) was placed in a Schlenk tube in an argon atmosphere glovebox. The tube was then capped and stirred at 132 °C for 48 h. After cooling to room temperature, the reaction mixture was poured into methanol. HCl (1N, 10 mL) was prepared and slowly added dropwise to the methanol solution. After the mixture was stirred for 20 min, the precipitate was collected by filtration and purified with Soxhlet extraction using methanol, acetone, hexane, and dichloromethane. The dichloromethane soluble fraction was concentrated and reprecipitated into methanol, yielding a purple solid (115 mg, yield 96%). ^1^H NMR (400 MHz, CDCl_3_) δ 9.22 (s, 1m H), 8.96 (s, 1m H), 4.19 (m, (4m+2n) H), 2.03 (m, (2m+n) H), 1.22 (m, (64m+8n) H), 0.81 (s, (12m+6n) H) ppm.

## Supporting Information Available

^1^H NMR and GPC charts, DSC and TGA curves, UV–vis-NIR absorption spectra in chloroform, glycerol contact angles, and photothermal properties of the polymer membranes in the wet state (PDF)
