# Supplementary material for: Enhanced Photothermal Property of NDI-Based Conjugated Polymers by Copolymerization with a Thiadiazolobenzotriazole Unit
Source: ACS Mater Au. 2023 Oct 26;4(1):82–91. doi: 10.1021/acsmaterialsau.3c00077 (PMC10786135; doi:10.1021/acsmaterialsau.3c00077)
Supplement: Supplementary file 1 — mg3c00077_si_001.pdf [file mg3c00077_si_001.pdf]

Supporting Information

for

Enhanced Photothermal Property of NDI-Based Conjugated Polymers by Copolymerization with a Thiadiazolobenzotriazole Unit

*Mingqian Wang, Chia-Yang Lin, Yoshimitsu Sagara, and Tsuyoshi Michinobu**

Department of Materials Science and Engineering, Tokyo Institute of Technology, 2-12-1 Ookayama, Meguro-ku, Tokyo 152-8552, Japan. E-mail: michinobu.t.aa@m.titech.ac.jp

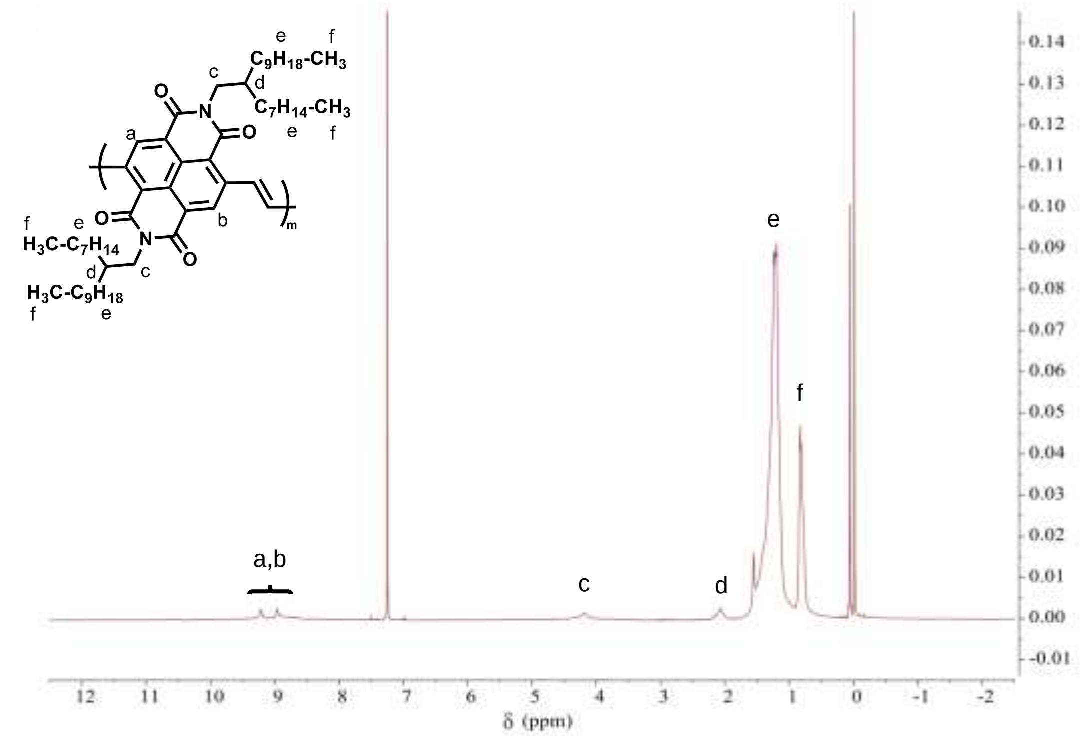

Figure S1 ^{1}H NMR spectrum of **P1** in $CDCl_3$.

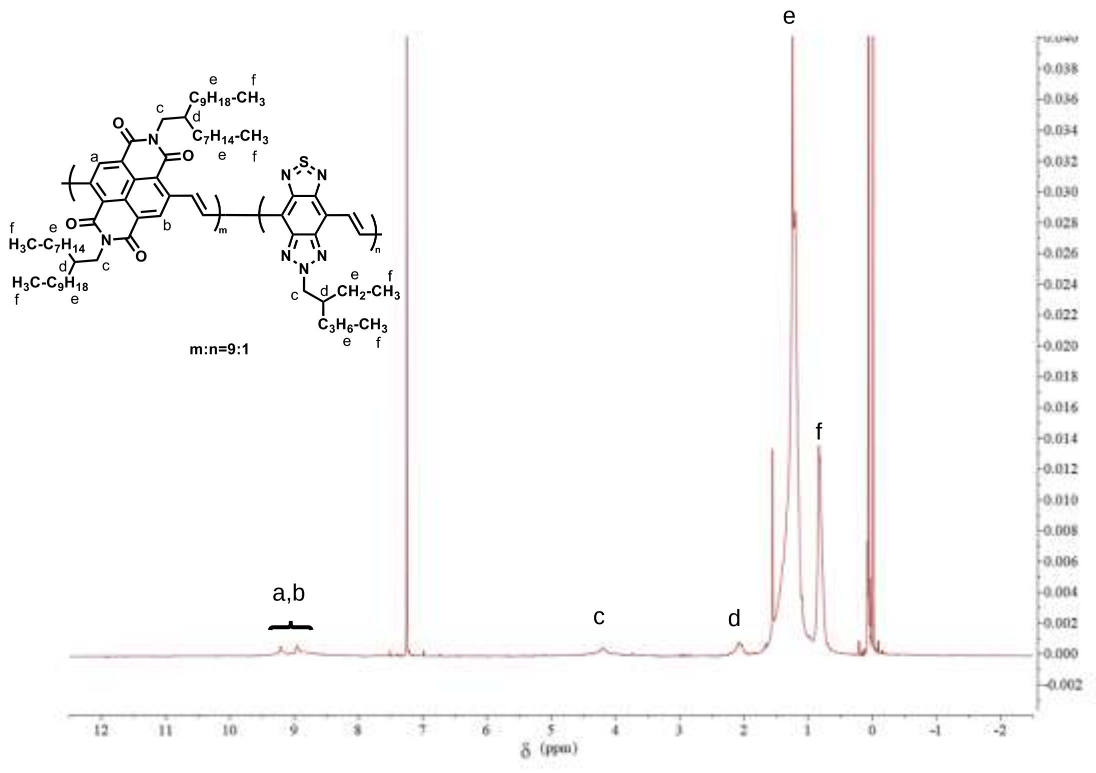

Figure S2 ^{1}H NMR spectrum of **P2** in $CDCl_3$.

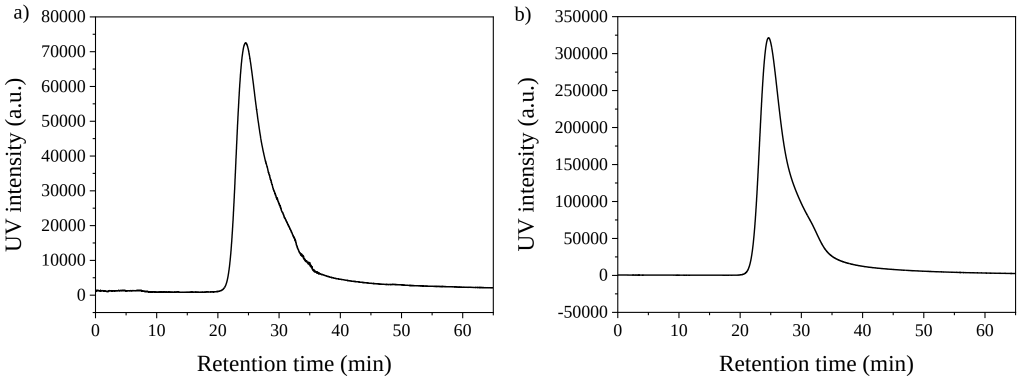

Figure S3 GPC charts of a) **P1** and b) **P2** (eluent: *o*-dichlorobenzene at 40 °C).

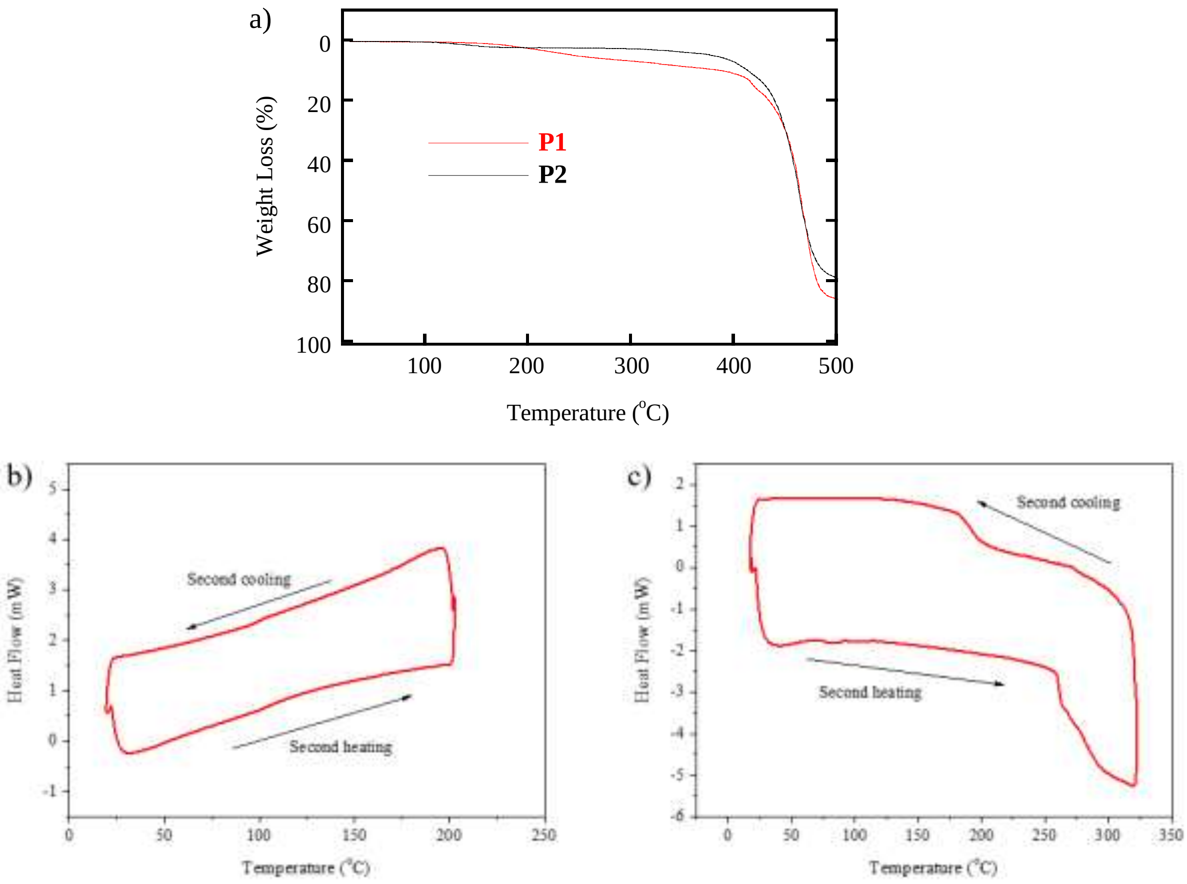

Figure S4 a) TGA curves of **P1** and **P2** at the heating rate of 10 °C min^{-1} under flowing nitrogen. DSC curves (second heating and cooling scans) of b) **P1** and c) **P2** at the scanning rate of 10 °C min^{-1} under flowing nitrogen

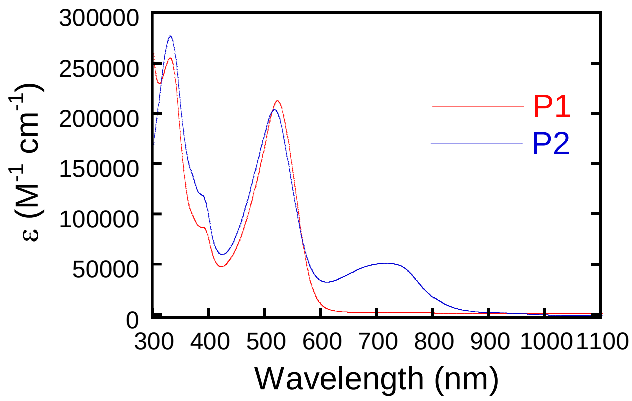

Figure S5 UV-vis-NIR absorption spectra of **P1** and **P2** in chloroform.

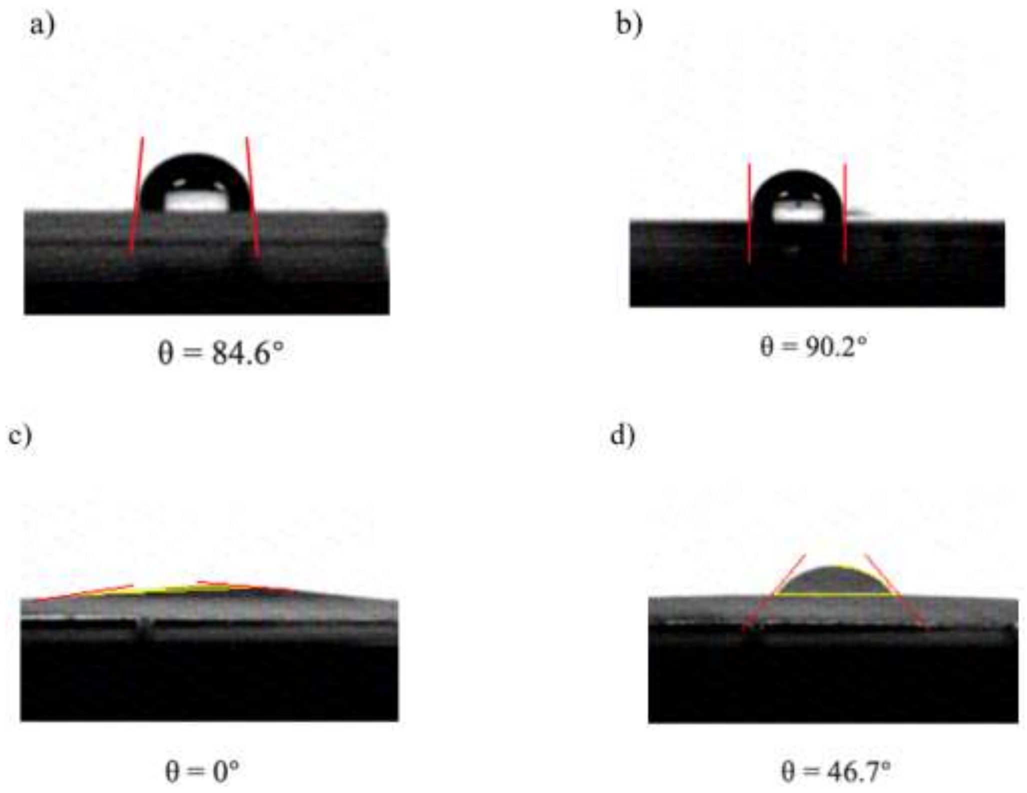

Figure S6 Glycerol contact angles on a) **P1** and b) **P2** films. c) Water and d) glycerol contact angles on a filter paper.

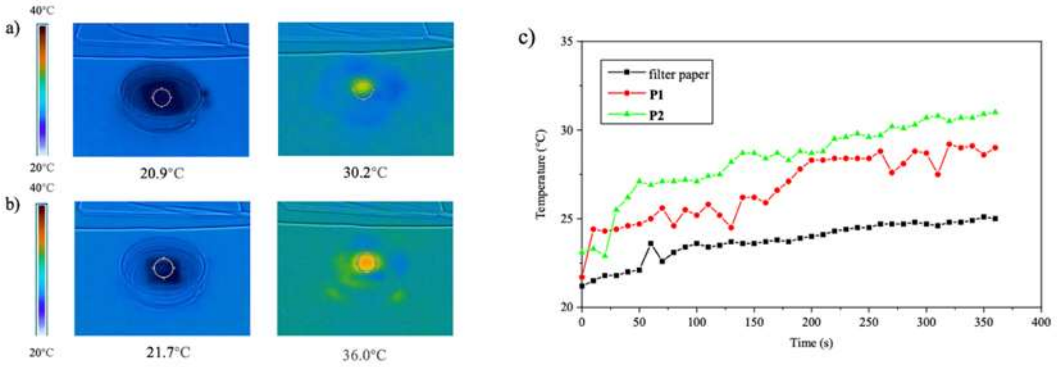

Figure S7 Thermal images of a) **P1** and b) **P2** membranes and c) time-dependent temperature changes of the **P1** and **P2** membranes and filter paper in the wet state under simulated sunlight irradiation.

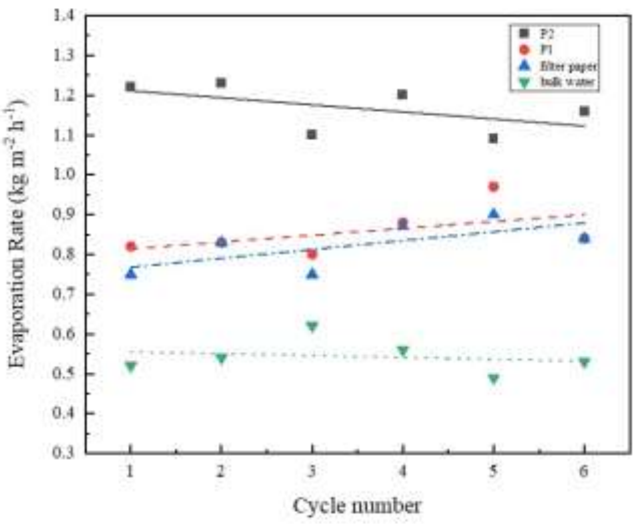

Figure S8 Repeated evaluation of evaporation rates (6 cycles) of the **P1** and **P2** membranes and filter paper.
